# The Epithelial-to-Mesenchymal Transition in Breast Cancer: Focus on Basal-Like Carcinomas

**DOI:** 10.3390/cancers9100134

**Published:** 2017-09-30

**Authors:** Monica Fedele, Laura Cerchia, Gennaro Chiappetta

**Affiliations:** 1 CNR—Institute of Experimental Endocrinology and Oncology, 80131 Naples, Italy; cerchia@unina.it; 2 Dipartimento di Ricerca Traslazionale a Supporto dei Percorsi Oncologici, S.C. Genomica Funzionale, Istituto Nazionale Tumori—IRCCS—Fondazione G Pascale, 80131 Naples, Italy; chiappettagennaro@gmail.com

**Keywords:** breast cancer, TNBC, EMT, tumor plasticity, molecular signaling, exosomes, miRNAs, αvβ3, differentiation therapy

## Abstract

Breast cancer is a heterogeneous disease that is characterized by a high grade of cell plasticity arising from the contribution of a diverse range of factors. When combined, these factors allow a cancer cell to transition from an epithelial to a mesenchymal state through a process of dedifferentiation that confers stem-like features, including chemoresistance, as well as the capacity to migrate and invade. Understanding the complex events that lead to the acquisition of a mesenchymal phenotype will therefore help to design new therapies against metastatic breast cancer. Here, we recapitulate the main endogenous molecular signals involved in this process, and their cross-talk with paracrine factors. These signals and cross-talk include the extracellular matrix; the secretome of cancer-associated fibroblasts, macrophages, cancer stem cells, and cancer cells; and exosomes with their cargo of miRNAs. Finally, we highlight some of the more promising therapeutic perspectives based on counteracting the epithelial-to-mesenchymal transition in breast cancer cells.

## 1. Introduction

Breast cancer is the most common cancer in women worldwide, and the fifth most common cause of death from cancer overall [1]. However, when we talk about breast cancer, as for most human cancers, we are referring to different tumors with respect to histopathological appearance, molecular alterations, presentation, and clinical outcome. According to most recent molecular classifications, breast carcinomas can be divided into at least six subgroups. These include normal-like (expression profile similar to noncancerous breast tissue); luminal A and B (generally estrogen receptor (ER)-positive tumors, with expression of epithelial markers; luminal B shows a higher Ki67 index and worse prognosis compared to luminal A); HER2 positive (overexpressing ERBB2 oncogene); basal-like (expressing basal cytokeratins and other markers characteristic of the myoepithelium of the normal mammary gland); and claudin-low (enriched in epithelial-to-mesenchymal transition (EMT) features, immune system responses, and stem cell-associated biological processes). Basal-like and claudin-low subtypes belong to the group of triple-negative breast cancer (TNBC), which are characterized by the lack of progesterone receptor (PR), ER and HER2 expression, and have high incidence of distant disease recurrence within three years of diagnosis, with a high frequency of visceral metastases [2]. A recent meta-analysis of a large cohort of TNBC cases allowed the subclassification of this group into at least four TNBC subtypes: luminal androgen receptor (LAR), mesenchymal (MES), basal-like immune-suppressed (BLIS), and basal-like immune-activated (BLIA) [3,4]. This subclassification is further supported by The Cancer Genome Atlas (TCGA) Program through mRNA, miRNA, DNA, and epigenetic analyses [5]. Considering the five main breast cancer subtypes, luminal A, luminal B, HER2, basal-like and claudin-low, a differentiation hierarchy that resembles the normal epithelial mammary developmental cascade has been proposed [6]. The claudin-low, which overlaps with the mesenchymal group, represents the most primitive tumors that are also the most similar to the mammary stem cells (MaSC). The following step in the mammary development is the luminal progenitor, which corresponds to the basal-like subtype. Then, a further development may lead a luminal progenitor/basal-like cell to the HER2 subtype, which represents the loss of the basal features and the acquisition of a luminal phenotype. Finally, the most differentiated groups are the luminal A and B subtypes [7]. Breast cancer patients with an undifferentiated phenotype similar to the normal MaSC have a worse prognosis compared with breast cancers with the more differentiated/luminal phenotype [6]. The process of dedifferentiation, which leads tumor cells to become increasingly more aggressive, is characterized in the last passage by an EMT process toward the claudin-low subtype. Indeed, the majority of death (90%) in breast cancer patients is caused by invasion and metastasis, two features related to the EMT [8]. The acquisition of EMT and stem cell-like features have been linked to each other [9,10], and have been associated with therapeutic resistance [5]. Indeed, breast cancer stem cells, which were originally isolated on the basis of the CD44^high^/CD24^low^/^Lin−^ immunophenotype [11], may be generated from breast cancer cells through the induction of an EMT, and EMT markers are expressed in stem-like cells isolated from mammary glands [9]. Cancer stem cells (CSCs) represent a small subpopulation of the tumor identified in most human tumors, including breast cancer [11]. These cells have self-renewal and tumor-initiating capabilities, which are determinant for the metastasization process [12]. A group of transcription factors playing critical roles during embryogenesis are also critical in the process of de-differentiation of the cancer cells. They induce EMT through transcriptional control of E-cadherin and include SNAIL1/2, ZEB1/2, TWIST1/2, FOXC1/2, TCF3, and GSC [13]. Among them, SNAIL and TWIST are able alone, if activated, to induce a mesenchymal/CSC phenotype in human immortalized human mammary epithelial cells [9,10]. Moreover, TWIST1, FOXC2, SNAIL1, ZEB2, and TWIST2 are overexpressed in stem-like cells isolated from primary breast carcinomas compared with more differentiated cancer cells [9].

## 2. The Role of EMT in Basal-Like Carcinomas

The EMT program associated with malignancy, invasion, and metastasis, also called EMT type 3 to distinguish it from those related to embryogenesis (type 1) and tissue regeneration (type 2), leads to a loss of cellular adhesion, changes in the polarization of the cell and cytoskeleton, migration, intravasation, survival in the vascular system, extravasation, and metastasis [8]. Therefore, it is believed to be a critical step in the progression of cancer toward a metastatic disease, even if the role for EMT in breast cancer metastases has been the matter of a recent debate on Nature [14,15]. Furthermore, EMT confers stem cell features contributing to chemoresistance and poor outcome [9]. Indeed, whereas neoadjuvant chemotherapy is associated with high pathologic complete response rates in basal-like carcinomas, metaplastic breast cancers (MBCs), which are aggressive TNBC tumors mostly characterized by EMT, are usually also chemoresistant and associated with worse outcomes [16]. The claudin-low subset is closely related to the MBC group by transcriptional profiling. Indeed, they are both characterized by the low expression of GATA3-regulated genes and genes involved in cell-cell adhesion, while are enriched of stem cell and EMT markers. However, they show differences in the presence of *PIK3CA* mutations and are therefore considered two different TNBC subgroups, even though they may have related cellular origins [17].

An intriguing capacity of the EMT process is that it is potentially reversible at any time by simply changing the expression of key molecular components. Accordingly, recent studies have indicated that mesenchymal-to-epithelial transition (MET), the reverse program of EMT, is observed in fibroblasts during the generation of induced pluripotent stem cells [18,19]. Further studies have shown that reprogramming factors introduced in cancer cells are able to attenuate their malignancy by letting them regain epithelial properties by MET [20]. Changes in cell phenotype between the epithelial and mesenchymal states are parts of the tumor progression process that leads tumor cells to disseminate in metastases. EMT is required for acquiring capability to migrate and invade, while MET is required to colonize the metastatic sites [21].

**Figure 1:**
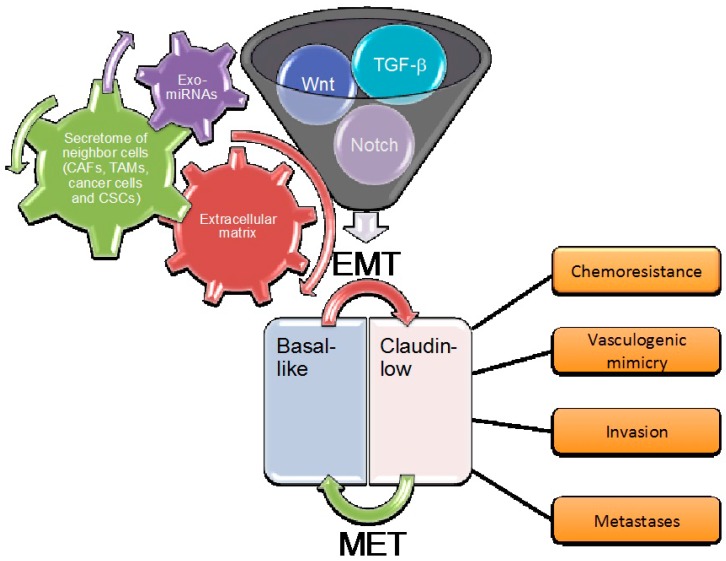
Contributing factors to the claudin-low phenotype and its impact on tumor behavior. The funnel encloses some of the main endogenous pathways involved in the epithelial-to-mesenchymal transition (EMT) process. The gear diagram indicates the various exogenous factors acting in a paracrine way on the endogenous EMT pathways. In the lower part of the scheme, the red upper arrow refers to the origin of claudin-low from basal-like carcinomas, whereas the green lower arrow depicts a possible reversion of the mesenchymal phenotype (mesenchymal-to-epithelial transition (MET) with the re-acquisition of the basal-like features. On the right, in orange, the cell functions resulted enhanced in the claudin-low phenotype as a consequence of the EMT induction.

This opens a potential challenge in that, by deeply dissecting all the pathways involved in the EMT program, we may discover new biomarkers and therapeutic agents for the most aggressive breast tumors. Indeed, different studies have shown that basal-like breast cancer, which is associated with mesenchymal features, is the most deadly subtype [6,22,23]. The acquisition of mesenchymal traits could be due to differences in the cells of origin, or the activation of oncogenes other than the paracrine induction of various EMT programs. However, how the mesenchymal phenotype is maintained is still a matter of intense investigation. There are both endogenous cell autonomous and exogenous non-cell autonomous signals concurring in the process of the EMT in breast cancer. The main endogenous pathways include those orchestrated by TGF-β, Notch, Wnt, Hedgehog, and receptor tyrosine kinases. Meanwhile, the exogenous signals include those coming from the extracellular matrix that act directly on the endogenous pathways, and those coming from the microenvironment, which act in a paracrine way. The latter includes the urokinase plasminogen activator system, the secretome of cancer associated fibroblasts, macrophages, cancer stem cells and cancer cells, and exosomes with their cargo of miRNAs (Figure 1). An integrated cross-talk among all these pathways, which adds further complexity to all of the process, has been observed.

## 3. Main Critical Endogenous Pathways of EMT in Breast Cancer Cells

**Figure 2:**
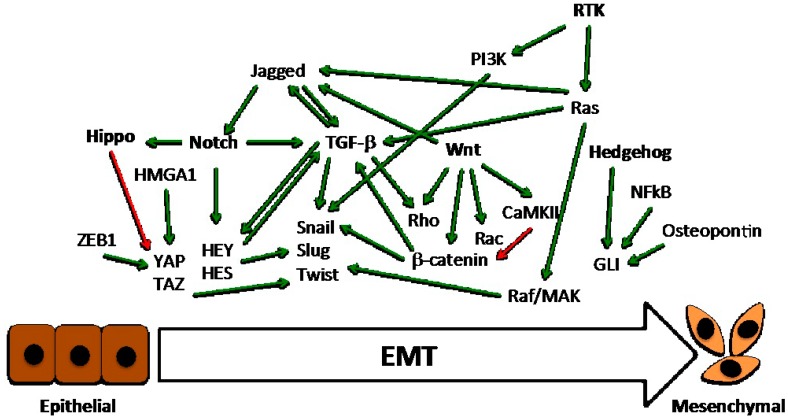
Schematic overview of the main critical endogenous pathways involved in breast cancer EMT. Green and red arrows indicate positive and negative regulation, respectively. The six pathways are in bold, and are further detailed in the text.

Six main critical pathways may be activated by means of genetic/epigenetic alterations, paracrine stimulation from neighbor cells, or direct interaction with ECM components in breast cancer cells, regulating their transition to a mesenchymal state (Figure 2).

### 3.1. The TGF-β Pathway

TGF-β signaling has a crucial and dual role in breast tumorigenesis. In early tumorigenic lesion, it has a tumor suppressive role due to its ability to induce growth inhibition. However, as cancer progress, it promotes tumor progression and metastasis mainly through the induction of EMT [24]. The TGF-β family of growth factors can initiate and maintain EMT in different cellular contexts [25]. They bind to cell surface receptors (types I and II) and form tight complexes with members of the Smad protein family, leading to their phosphorylation [26]. Phosphorylated Smads associate with cytoplasmic Smad4 and translocate to the nucleus where Smad complexes control transcription of target genes [27]. Moreover, TGF-β may alter the cell surface protein complex structure and expression directly through its receptor complex, independently from nuclear gene regulation. Indeed, TGF-β ligand binding enables type II TGF-β receptor kinase, which is associated with occludin at tight junctions, to phosphorylate Par6 [28]. This protein-protein interaction is direct and independent of Smad proteins [28]. The phosphorylation of Par6 allows it to recruit Smurf1, which in turn leads to the ubiquitination and degradation of RhoA [28], a small GTPase family member responsible for stress fiber formation and for the maintenance of apico-basal polarity and junctional stability [29,30]. Other members of the Rho family, which regulate the cytoskeletal remodeling associated with the gain of cell motility, focal adhesions, and adherens junction formation; and with the interactions between adherens junctions and actin filaments, are also activated by EMT signaling events induced by TGF-β [31,32,33,34]. Thus, Rho family GTPases and their regulatory molecules (GEFs and GAPs) exert key roles in epithelial plasticity, and are crucial effectors of TGF-β-induced EMT [30].

The TGF-β pathway cross-talks with different pathways for the induction and maintenance of the EMT phenotype. In mammary epithelial cells, oncogenic Ha-Ras activation promotes EMT through autocrine production of TGF-β [35], and activation of the Raf/MAK pathway is required for metastatic features of EMT in vivo [36]. Another cross-talk of the TGF-β signaling in EMT has been described with the Notch pathway. On one hand, a subset of Notch target genes, including HEY1, HEY2, HES1, and HES5, and the Notch ligand Jagged1, are induced by TGF-β at the onset of EMT in a panel of epithelial cells from the mammary gland, kidney tubules, and epidermis. On the other hand, the silencing of HEY1 or Jagged1, as well as the chemical inactivation of Notch, inhibits TGF-β-induced EMT [37]. Finally, a TGF-β crosstalk with the Wnt pathway has been also described. In a mammary gland model, β-catenin transcriptional activity leads to the activation of autocrine TGF-β signals, which synergize with the Wnt signaling to maintain the mesenchymal phenotype [38].

### 3.2. The Wnt Pathway

The Wnt pathway plays a critical role in the development and progression of breast cancer [39]. In many human cancers, including breast cancer, it promotes cell migration and EMT through the stabilization of Snail [40]. Consistently, Wnt signaling up-regulates the transcription factors Slug and Twist [41,42]. The Wnt pathway is composed of two distinct branches: the canonical and non-canonical pathways. In the canonical pathway, Wnt protein binds to the cell surface receptor Frizzled, which forms a complex with the coreceptors Lrp5 and 6 to promote Axin binding to Dishevelled. This leads to stabilization and translocation to the nucleus of β-catenin. There, β-catenin interacts with transcription factors of the Tcf/Lef family to activate the expression of several genes [43]. β-catenin translocation after Wnt activation can also be mediated through the sequestration of GSK3, the protein that phosphorylates and destabilizes β-catenin, inside multivesicular endosomes [44]. The secreted Frizzled-related protein SFRP1, which is homologous to the extracellular cysteine-rich domain of Frizzled, prevents Wnt ligands from binding, thereby acting as a negative regulator of Wnt signaling [45]. Another secreted protein, DKK1, also inhibits the canonical Wnt pathway by binding directly to the Lrp5/6 coreceptors [46]. Both SFRP1 and DKK1 are frequently silenced by methylation in many human cancers, including breast cancer [47]. The non-canonical, β-catenin-independent Wnt pathways include the planar cell polarity pathway, which regulates cellular organization and polarity through cytoskeletal organization, and the Wnt/Ca^2+^ pathway, which results in a release of intracellular calcium regulation of cell adhesion and migration [48]. In the planar cell polarity pathway, Wnt binds the Frizzled and coreceptors ROR and Ryk. This activates Rho and Rac, which in turn activate Rho-associated protein kinase (ROCK) and c-Jun N-terminal kinase (JNK), respectively, leading to actin polymerization. In the Wnt/Ca^2+^ pathway, Wnt ligands bind to Frizzled, which interacts with G proteins and Dishevelled, thereby activating cGMP-specific phosphodiesterase or phospholipase C, which cause the release of intracellular calcium. Ca^2+^ activates CaMKII, which in turn regulates cell adhesion and migration through activation of nuclear factor of activated T cells (NFAT) [48]. This non-canonical Wnt pathway antagonizes canonical Wnt/β-catenin signaling by activation of Nemo-like kinase, which phosphorylates TCF4 and prevents the β-catenin–TCF4 complex from binding to DNA [49]. 

Using an orthotopic model of human breast cancer metastasis to lung, DiMeo et al. demonstrated that Wnt signaling is required for tumor formation and metastases, and is necessary for the capacity of cancer cells to self-renew and to maintain the dedifferentiated EMT state, thus providing a molecular link among self-renewal, EMT, and metastasis in breast cancer [50]. More recent studies have shown that the nuclear accumulation of β-catenin is required for TNBC development by controlling tumor-associated properties, such as migration, stemness, anchorage-independent growth, and chemosensitivity, thus suggesting that canonical Wnt signaling is a major driving force in breast cancer [51]. Other studies have also implicated the dysregulation of non-canonical Wnt signaling pathways in the highly metastatic behavior of TNBC cells and CSCs, specifically through aberrant JNK activation [52].

### 3.3. The Notch Pathway

The Notch ligand-receptor interaction system in vertebrates comprises four receptors (Notch1–Notch4) and five ligands from the Delta and JAG/Serrate (DSL) families: JAG1 and 2 and Delta-like (DLL)-1, 3, and 4 [53]. The interaction between ligand and receptor triggers a series of proteolytic cleavages that release the Notch intracellular domain (NotchIC), allowing it to translocate into the nucleus. Within the nucleus, NotchIC binds to the transcriptional repressor CSL, resulting in the derepression and coactivation of target genes, which regulate various cellular processes. Interestingly, in the development of cancer, Notch may act as either an oncogene or a tumor suppressor gene, depending on the tumor type [54]. In breast carcinoma pathogenesis, Notch signaling and its cross-talk with various pathways—Notch has been reported to be activated downstream of Ras and Wnt in the promotion of mammary tumors [55,56]—play an important role in cell growth, migration, invasion, angiogenesis, and metastasis [57]. Its activation correlates with poor prognosis and poor patient survival [58], induces EMT [59], and promotes the malignant features of breast cancer [60]. JAG1-mediated activation of Notch in breast epithelial cells induces EMT through the induction of Slug and the subsequent repression of the cell–cell adhesion protein E-cadherin, and EMT plays a crucial role in promoting metastases in tumor xenografts exhibiting ligand-induced Notch signaling [59]. Consistently, mammary-specific overexpression of constitutively active Notch1, Notch3, or Notch4 in mice leads to the formation of aggressive, metastatic breast tumors [61,62], and Notch signaling plays a crucial role in stemness [63]. On the other hand, it has been also recently reported that Notch3, inhibits EMT in breast cancer by activating the Hippo pathway, but Notch1 does not [64].

### 3.4. The Hippo Pathway

The Hippo tumor suppressor pathway consists of a large network of proteins that play important regulatory functions during organ development and regeneration. The core components of this network include a kinase and a transcription module. The kinase module includes the MST1/STK4 and MST2/STK3 protein kinases, the large tumor suppressor proteins LATS1/2, and the adaptor proteins SAV1 and MOB1A/B. This module contributes to the LATS1/2-dependent phosphorylation of yes-associated protein (YAP) and tafazzin (TAZ), which are members of the transcriptional module. The phosphorylation of YAP and TAZ represses their activity by creating 14-3-3 binding sites that cause their cytoplasmic accumulation and proteasome degradation [65]. In breast cancer cell lines, the phosphorylation and activation of YAP, which enhance cell motility and invasiveness, are dependent on the HMGA1-CyclinE2 axis [66]. YAP and TAZ activity have been shown to be increased in basal breast cancers that show a stem-cell-like phenotype [67], and YAP overexpression has been reported to promote the EMT of cultured breast cancer cells [68]. Furthermore, YAP and TAZ activity were increased in high-grade metastatic breast cancer specimens compared with low-grade non-metastatic breast cancer [67], and TAZ is required for the metastatic activity and chemoresistance of breast cancer stem cells [69]. Still in breast cancer, signal transduction from the metastasis suppressor leukaemia inhibitory factor receptor (LIFR) was shown to sequester and inactivate YAP. Therefore, the loss of LIFR expression could be one mechanism that results in YAP or TAZ hyperactivation during the metastasis of breast cancers [70]. Another mechanism might be the loss of E-cadherin, which causes YAP and TAZ derepression in metastatic breast cells [71]. More recently, the Hippo pathway inhibition has been shown to be required for the increased migratory and invasiveness ability of breast cancer cells in twist-mediated EMT [72]. Also, the EMT-inducing transcriptional repressor ZEB1 has been shown to directly interact with and activate the Hippo pathway effector YAP [73], and the E3 ubiquitin-protein ligase Itchy homolog (ITCH) has been shown to enhance EMT in breast cancer by negatively regulating LATS1, and therefore increasing YAP activity [74]. All together, these findings support a central role of the Hippo pathway in counteracting EMT and metastases in breast cancer. 

### 3.5. The Hedgehog Pathway

A growing body of literature supports the role of the stem cell renewal Hedgehog (Hh) pathway in breast cancer [75]. The Hh pathway plays a key role in embryonic development, and regulates stem cell renewal and tissue homeostasis [76]. It involves a signaling cascade starting from the three secreted proteins Sonic (SHH), Indian (IHH), and Desert (DHH) Hedgehog, and the two trans-membrane receptors Patched and Smoothened. It then terminates with the activation of the three glioma-associated oncogene (GLI) transcription factors, GLI1, GLI2, and GLI3, which can function as either activators or repressors of transcription [77]. As for Wnt signaling, canonical and non-canonical pathways have been described for the Hh pathway, too. The canonical pathway is the above-described signaling that through Hh/receptor binding leads to GLI activation, whereas non-canonical Hh pathways are considered either a cellular response mediated by Patched and Smoothened but independent from GLI [78], or GLI activation independent from Hh ligand/receptor binding [79].

Evidence supporting the contribution of the Hh pathway to EMT in breast cancer has been reported different studies. Using a high throughput inhibitor screen, Colavito et al. identified the high expression of GLI1 as a critical determinant of breast cancer cell lines that have undergone an EMT [80]. Their work also showed the importance of the Hh pathway in the maintenance of CSC features, and uncovered a cross-talk between NFkB and GLI1 [80]. Other studies reported that the non-canonical activation of GLI1 by the inflammatory cytokine osteopontin or hypoxia results in the induction of EMT, drug resistance, and invasion capabilities in breast cancer cell lines [81,82]. Moreover, the development of mammary tumors by the conditional expression of GLI1 in experimental mouse models further supports the implication of the Hh pathway in EMT-mediated breast tumorigenesis [83].

### 3.6. Pathways Emanating from Receptor Tyrosine Kinases

Receptor tyrosine kinases (RTKs) have a crucial role as sustainers and effectors of EMT in a variety of tumors, including breast cancer [84]. The activation of RTKs occurs through homodimerization induced by ligand binding, or ligand independent mechanisms, including transactivation or heterodimerization with other RTKs or non-RTKs receptors [85]. Growth factors such as EGF, FGF, IGF, and PDGF, stimulate RTKs to initiate intracellular signaling (including those mediated by Ras, PI3K, Src, and ILK), which ultimately could promote the expression of EMT-inducing transcription factors such as Snail1/2, ZEB1/2, and Twist, contributing and/or regulating EMT [84]. Some RTKs, as PDGFRβ [3] and Axl RTKs [86], are emerging as mesenchymal/stem cell-specific markers in breast cancers. However, whether RTKs induces EMT or whether EMT induces receptor expression is still an open debate [87].

Importantly, many of the signaling cascades (including various branches of Mitogen-activated protein kinase, Rho-like GTPase, and PI3K/AKT pathways) induced by TGF-β, a primary inducer of EMT, are also induced by RTKs in response to ligand binding, and a complex cross-talk of oncogenic signaling has been implicated in EMT [88]. It has been reported that mammary cancer metastasis is strongly promoted by an autocrine PDGF/PDGFR loop, which is established as a consequence of TGF-β-induced EMT [89]. Also, RTK-dependent signaling has not only an established role in the induction of classical EMT transcription factors, it also regulates the deposition of several ECM components and integrin binding to ECM, thus activating intracellular cascades that mediate EMT (see below). 

## 4. Role of the Extracellular Matrix

It has been recently shown that breast cancer cell lines representative of the mesenchymal/claudin-low subtype have the capability to undergo endothelial transdifferentiation forming spiderweb-like networks. This phenomenon is known as vascular mimicry (VM), which provides the blood supply for tumor growth and promotes metastasis with mechanisms distinct from classical angiogenesis [90]. The VM process is essentially dependent on cell-matrix interaction mediated by integrins, which are cell surface adhesion molecules representing the main receptors by which the cells bind to and respond to extracellular matrix (ECM) components. Among them, integrin αvβ3 expression strongly correlates with tumor invasion, EMT, and metastases of highly aggressive cancers [91,92]. Different RTKs have been shown to associate with αvβ3, thus promoting many aspects of tumor progression, including VM, migration, invasion, and metastases. In response to matrix, integrin αvβ3 forms a complex with the epidermal growth factor receptor (EGFR) on the surface of TNBC claudin-low MDA-MB-231 and BT-459 cell lines, which are crucial for VM [92]. This interaction allows integrin to adopt a conformation competent for binding to the ECM, which is required for VM. A similar role is played by the PDGFRβ [93], and other examples of cross-talk between integrins and RTKs, also based on a physical interaction among them, have been reported [91]. Indeed, the high expression of integrin αvβ3 has been recently shown to be a marker of breast carcinomas with stem-like features, and high resistance to tyrosin kinase inhibitors [94]. In many cases, the cross-talk between integrins and RTKs leads to the degradation or recycling of the receptor, thus regulating the engagement of matrix ligands [95]. It has been also reported that the association of αvβ3 with different RTKs, including PDGFRβ and VEGFR2, in the presence of ECM ligands, augments the ability of RTKs to respond to their growth factors, thus resulting in the induction of cell proliferation and migration [96]. Another component of the ECM, periostin (POSTN), is induced in breast cancer metastases, where it has been found to play a critical role in their development through the maintenance of CSCs [97]. To this aim, POSTN interacts with Wnt ligands, boosting Wnt signaling, which in turn control stem cell maintenance [97]. Also, the matrix metalloproteinases (MMPs), which degrade and modify the ECM as well as cell-ECM and cell-cell contacts, facilitating detachment of epithelial cells from the surrounding tissue, are upregulated in breast cancer, where they stimulate tumorigenesis, cancer cell invasion, and metastasis by activating EMT [98].

## 5. Paracrine Mechanisms

Cancer cells secrete proteins that modify the extracellular milieu, acting as autocrine and paracrine stimulatory factors, and have a relevant role in cancer progression [99]. This secretome, which is released by the cells via different pathways [100], contributes to EMT, the metastatic spreading of cancer cells, and the maintenance of CSCs. Also, cancer-associated fibroblasts (CAFs) assist tumor invasion and promote the oncogenic transformation of surrounding epithelial cells by secreting numerous pro-tumorigenic factors [99,101]. In breast cancer cells, CAFs promote aggressive phenotypes through EMT induced by paracrine TGF-β1 [102]. They may also originate by differentiation of bone marrow-derived mesenchymal stem cells, which migrate to the tumor site and contribute to the tumor microenvironment [103,104]. They also promote the aggressiveness of TNBC cell lines that are evaluated as capable of migrating, invading, and acquiring stemness markers [105]. CSCs themselves have their own secretome, which is different from that of the bulk tumor cells and their derived differentiated cancer cells. Different studies indicate a role for CSC-secreted TGF-β in the transformation of breast cancer cells to CSCs, and in the TGF-β-mediated metastasis of the cancer cells tissues [99]. Indeed, breast cancer cells have shown a gene signature that is consistent with the activation of TGF-β signaling. This signature includes the elevated expression of TGF-β and its receptors in CD44^+^/CD24^−^ CSCs compared with the CD44^−^/CD24^+^ non-stem cells [106]. Furthermore, in vitro treatment of human mammary epithelial cells with TGF-β has been shown to give rise to CD44^+^/CD24^−^ CSCs through induction of the EMT [9]. Also, it has been recently reported that in mammary glands, tumor CSCs activate CAFs via the paracrine activation of Hedgehog signaling, thus inducing the CAFs’ secretion of factors that promote the expansion and self-renewal of CSCs [107]. Finally, a paracrine loop between tumor cells and tumor-associated monocytes and macrophages (TAMs) has been described in mammary tumors to allow tumor cell migration and CSC niche support [108,109]. In the latter case, the EMT program mediates the physical interactions of CSCs with TAMs by receptor-counter-receptor binding, thus activating signalings in CSCs that culminate with the secretion of cytokines sustaining the stem cell fate [109]. The reciprocal reprogramming of both the tumor cells and the surrounding cells and tissue structures not only guides invasion, it also generates diverse modes of dissemination [110]. Some of the factors that are necessary for the induction of different EMT pathways in breast cancer cells are secreted by cells that are in an epithelial state and fail to act in an autocrine way, but act in a paracrine way on neighbor cancer cells. Then, once cells have passed through an EMT, they maintain the resulting mesenchymal/CSC state by cell-autonomous autocrine loops [111]. An autocrine PDGF/PDGFR loop, which contributes to the maintenance of EMT, is established in breast cancer cells as a consequence of TGF-β signaling [89].

One of the most well-studied paracrine mechanisms involved in the early metastatic step of breast cancer is the urokinase plasminogen activator (uPA) system, composed by the protease uPA and its receptor uPAR, which converts the plasminogen in plasmin. Plasmin in turn degrades—either directly or indirectly through the activation of matrix metalloprotease—several ECM proteins, including fibronectin, laminin, and others. This releases growth factors that stimulate proliferation, migration, invasion, and metastasis upon binding to their cognate receptors [112,113]. Moreover, the uPA/uPA complex cooperates with integrins, G-protein coupled receptors, caveolins, and lipids rafts for signal transduction. Indeed, uPA and its inhibitor PAI-1 are markers of poor prognosis and metastases in primary breast tumors [114,115], and evidence has been reported that the uPA system facilitates breast cancer metastases by several mechanisms [116].

### Exosomes and microRNAs

Several cellular components of the tumor microenvironment and cancer cells secrete exosomes that function in an autocrine or paracrine manner to promote many aspects of cancer cells. These aspects include angiogenesis, invasion, proliferation, and contribution to cancer cell plasticity by regulating EMT in the tumor microenvironment [117]. They are small vesicles that originate from the plasma membrane and released from the cell in the extracellular milieu. They contain a wide variety of biological active material that they can exchange with neighboring cells, thus enabling a potent mode of intercellular communication [118,119]. Unlike soluble factors secreted by cells, exosomes carry a concentrated group of functional molecules, provide protection to the transported molecules, and serve as intercellular communicators not only locally, but also systemically [117]. This group of functional molecules may include oncoproteins and oncomiRNAs. The oncogenic message may be transferred by exosomes in different ways: (i) by releasing ligands in the extracellular milieu; (ii) by fusion with the plasma membrane of recipient cells; and (iii) by endocytosis [117]. Luga et al. observed that Wnt containing exosomes derived from CAFs promoted motility and metastasis by activating Wnt signaling in recipient breast cancer cells [119]. Similarly, exosomes derived from mesenchymal stem cell and macrophages promoted the migration and/or invasion of breast cancer cell lines via activation of Wnt signaling [120,121]. Meanwhile, paracrine Wnt10b transported by exosomes released by CAFs can promote cancer progression via EMT induced by the canonical Wnt pathway [122]. On the other hand, exosomes from breast cancer cells can convert adipose tissue-derived mesenchymal stem cells into myofibroblast-like cells [123]. Exosomes are also involved in mediating hypoxia-induced EMT. Specifically in breast cancer cell lines, the induction of hypoxia has been shown to result in the release of an increased number of exosomes, which contain miR-210 [124]. This could play a role in promoting tumor progression in response to hypoxia, as miR-210 can promote endothelial cell tubulogenesis [125].

Several miRNAs have been implicated in the regulation of EMT in cancer [126], and exosome-mediated exchange of miRNAs (exo-miRNAs) between cells has been reported in recent years [127]. MiR-223, a miRNA transported from exosomes released from IL-4-activated macrophages to breast cancer cells, promote breast cancer cell invasion via modulation of the β-catenin pathway [128]. Therefore, tumor and stromal cells can regulate EMT and metastasis through the exosome-mediated delivery of proteins and miRNAs. Other miRNAs related to EMT in breast cancer include either negative regulators (miR-200 family, miR-34 family, miR-497, miR-125b, miR-206, miR-30a, miR-138, miR-195, miR-143, miR-671-5p, miR-153, and miR-300), or positive regulators (miR-10b, miR-21, miR-155, miR-9, miR-29a, miR-103/107, miR-181b-3p, miR-221/222, miR-183/96/182, miR-373, and miR-100). For a recent detailed review of endogenous miRNAs and networks that participate in breast cancer, see elsewhere [129]. Interestingly, different miRNAs may cross-regulate the tumor EMT process. It has been shown that miR-103/107 induces EMT in breast cancer by downregulating miR-200, which targets the E-cadherin negative regulators ZEB1 and ZEB2 [130,131]. Further, a network involving PDGFRβ, miR-9, miR-200, and EMT has been described in mesenchymal TNBC subtypes. Indeed, it has been shown that the induction of miR-9 by PDGFRβ stimulation strongly increases the VM of TNBC cells, whereas ectopic expression of miR-200 causes the reduction of PDGFRβ levels by suppressing ZEB1, and in turn inhibits vasculogenic properties [92].

## 6. Therapeutic Perspectives

Highly aggressive breast cancer subtypes, such as the claudin-low group, are clinically resistant to chemotherapy due to their enrichment in CSCs. The association between the EMT program and the CSC state represents an attractive opportunity for drug development that is only recently starting to be experimentally proven. A differentiation therapy that is based on the induction of a MET is indeed a possible road to tread: activation of PKA leads to MET and loss of tumor-initiating ability in breast cancer cells [132]. However, a caveat of using such a MET-induced differentiation therapy is the observed requirement of a MET to complete the colonization stage of the metastasis cascade. Consequently, the induction of a MET might inadvertently support the process of metastatic colonization at distant sites [133]. Weinberg’s group has recently employed a therapeutic approach that involves the differentiation of CSCs to their non-stem cell counterparts through the induction of a MET. They showed that the induction of a MET as a form of differentiation therapy may improve the response of advanced carcinomas to chemotherapy and prevent their progression to metastasis [134]. A growing list of compounds that reverse EMT in breast cancer has been used in preclinical studies. Through using erbulin, a non-taxane microtubule dynamics inhibitor, for seven days on TNBC cells, Yoshida et al. demonstrated that the treatment induced MET while resulting in decreased in vitro migration and invasiveness, as well as decreased numbers of lung metastasis, when assessed in an in vivo experimental metastasis model [135]. Similar results have been obtained using luteolin, a natural flavonoid compound [136]; diallyl disulfide, an important garlic (Allium sativum) derivative [137]; and mangiferin, a naturally occurring glucosylxanthone [138], which suggests that these compounds could be potential therapeutic candidates for the treatment of advanced or metastatic breast cancer.

As well as being an essential step in tumor metastases, EMT could also be induced under the selective pressure of clinical cytotoxic drugs. To solve this problem, Fan et al. have synthesized multi-functional epigallocatechin gallate/iron nano-complexes (EIN) as a versatile coating material to improve conventional therapies. They showed in vitro that this strategy could eliminate EMT-type cancer cells, and in vivo studies revealed that EIN inhibits the EMT process and enhances the therapeutic effect of conventional chemotherapy, thus preventing drug chemoresistance [139]. Further, a new approach, the ABC7 regimen (Adjuvant for Breast Cancer treatment using seven repurposed drugs), has been recently proposed for metastatic breast cancer. In addition to the current standard treatment with capecitabine, ABC7 uses an ensemble of seven already-marketed noncancer treatment drugs to block different EMT signaling pathways, as a way to make current traditional cytotoxic chemotherapy more effective. However, it has not yet been experimentally tested for its safety and effectiveness [140].

Another therapeutic strategy against EMT may be using monoclonal antibodies or oligonucleotide aptamers that are able to bind to cancer cell surface proteins and disrupt their attachment to the extracellular matrix via integrins. We recently provided evidence that the anti-EGFR CL4 aptamer impairs the integrin αvβ3-EGFR complex on TNBC cells grown on Matrigel or subcutaneously injected in nude mice to form tumors. This causes the inhibition of integrin binding to matrix and, in turn, VM in vitro and in vivo [92]. A similar effect can be obtained by Transtuzumab, a monoclonal antibody against HER2, which causes the loss of integrin αvβ6 and HER2 in breast cancer xenografts [141]. Another interesting approach involving aptamers consists in the selective delivery of therapeutic siRNAs or drugs to breast tumors by using aptamers as delivery agents. In this context, aptamer targeting EpCAM was shown to inhibit CSCs when linked to siRNAs against *PLK1*, a kinase required for mitosis, and cause tumor regression when injected in the TNBC xenograft model [142].

Finally, strategies to interfere with the loading or delivery of tumor-promoting exo-miRNAs or to replenish tumor-suppressive miRNAs via exosomal delivery are under investigation [143], and they can potentially be employed to deliver either miRNAs that negatively regulate EMT, or antagomirs against miRNAs that positively regulate EMT in breast cancer cells. Functional studies showed that the inhibition of miR-23a suppressed the TGF-β1-induced EMT, migration, invasion, and metastasis of breast cancer cells, both in vitro and in vivo [144]. Other studies reported that: miR520c could inhibit breast cancer EMT by targeting STAT3 [145]; miR-10b antagomirs inhibit metastasis in a mouse mammary tumor model [146]; and that miR200c expression significantly enhanced the chemosensitivity and decreased the metastatic potential of a p53(null) claudin-low tumor model [147], and restored trastuzumab sensitivity while suppressing invasion of breast cancer cells [148]. However, all of these studies did not use exosomes to deliver the miRNAs. A recent study showed that the delivery of miR-134 by exosomes in TNBC cells caused the reduction of cellular migration and invasion [149]. This gave a proof of concept of a possible exo-miR therapy. Furthermore, docosahexaenoic acid alters breast cancer exosome secretion and microRNA contents, including EMT-inducing miRNAs, in breast cancer cells [150], which supports its use for a breast cancer therapy aiming to counteract the paracrine effects of exo-miRNAs.
